# Real-world adherence for direct oral anticoagulants in a newly diagnosed atrial fibrillation cohort: does the dosing interval matter?

**DOI:** 10.1186/s12872-019-1033-3

**Published:** 2019-03-19

**Authors:** Phuong N. Pham, Joshua D. Brown

**Affiliations:** Department of Pharmaceutical Outcomes & Policy, University of Florida College of Pharmacy, 1225 Center Drive, HPNP #3320, Gainesville, FL 32610 USA

**Keywords:** Atrial fibrillation, Anticoagulants, Adherence, Switching

## Abstract

**Background:**

Differences in adherence may represent drug properties (e.g. dosing interval) or patient experiences while on treatment. Adherence to direct oral anticoagulants (DOACs) in nonvalvular atrial fibrillation (NVAF) is important to maintain effectiveness over the course of treatment.

**Methods:**

This was a retrospective cohort study using 2009–2015 Truven Health MarketScan Databases. New initiators of dabigatran, rivaroxaban, and apixaban with NVAF were identified. Twelve months of continuous enrollment before treatment was required to assess demographics and medical history. Proportion of days cover (PDC) was used to measure adherence at 3, 6, 9 and 12-month. Gaps in therapy and treatment switches were also evaluated. Logistic regression was used to compare high adherence (PDC ≥0.80).

**Results:**

A total of 14,864 dabigatran, 16,005 rivaroxaban, and 8078 apixaban users were identified. Apixaban users had the highest adherence overall, with mean PDC at 3, 6, 9, and 12-months of 0.83, 0.76, 0.72, and 0.69, while dabigatran had the lowest adherence of 0.78, 0.67, 0.61, and 0.57. Adherence to DOACs increased with increased stroke risk scores. Adherence was also higher when first days supplied was > 30 days compared to 30 days and when filled via mail order pharmacies. Switching was highest among dabigatran users. Apixaban users were the most likely to have high adherence versus dabigatran (OR = 1.73, 95% CI = 1.60–1.88) and versus rivaroxaban (OR = 1.24, 95% CI = 1.14–1.34) at 12-months.

**Conclusions:**

Apixaban users had the highest overall adherence despite twice-daily dosing versus once-daily dosing for rivaroxaban. These findings can be useful for formulary decision-making and when assessing treatment options.

## Background

Direct-acting, non-vitamin K antagonist, oral anticoagulants (DOACs) are widely utilized for stroke prevention in non-valvular atrial fibrillation (NVAF) [1]. Dabigatran was first introduced in 2010, followed by rivaroxaban in 2011, and apixaban in 2012. Rivaroxaban, dabigatran, and apixaban have been shown to be non-inferior or superior to warfarin in both efficacy to lower the risk for thromboembolism as well as for bleed-related safety outcomes [2–4]. Edoxaban was also introduced in 2015 [5] and betrixaban was approved in 2017 only for venous thromboembolism indications.

Indirect treatment comparisons and observational comparative effectiveness studies have shown that while relative differences between DOACs are small, apixaban and dabigatran appear to be more effective and/or safer than rivaroxaban [6–8]. However, despite potential for lower net benefit, arguments can be made for using rivaroxaban to improve treatment adherence given a once daily dosing interval (versus twice daily for others) as well as the avoidance of certain side effects (e.g. dyspepsia with dabigatran) - all which may influence treatment selection [9, 10]. Thus, the relative differences in adherence between rivaroxaban, dabigatran, and apixaban may be an important proxy for patient behaviors, preferences, side effects, effectiveness and safety among these alternative therapies and are an important additional consideration along with estimates of the efficacy of each medication in order to extrapolate their real-world effectiveness [11–14].

Understanding the differences in adherence for each therapy can help inform treatment choices by patients and physicians as well as formulary decision-making and health technology assessments given adherence can be tied to treatment outcomes. The U.S.-based Pharmacy Quality Alliance (PQA), which has health plan quality metrics that have been adopted by a large number of health systems, managed care organizations, as well as by the Centers for Medicare and Medicaid Services (CMS), has endorsed a quality measure regarding adherence to DOACs for all indications (NVAF stroke prevention, venous thromboembolism prevention and treatment) [11, 15]. This study sought to update the evidence from a previous studies on the adherence to DOACs in a cohort of commercially insured, newly diagnosed NVAF patients in the United States using the most contemporary data available, 2010–2015 [16, 17].

## Methods

### Cohort selection and data source

This was a retrospective cohort study using the Truven Health MarketScan Commercial Claims and Medicare Supplemental Databases, which represent the medical and pharmacy healthcare claims of 20–40 million individuals annually. The data for this study were obtained from 2009 to 2015 to capture the availability of DOACs in the U.S. market. The University of Florida Institutional Review Board approved this study as exempt.

All new users of dabigatran, rivaroxaban, and apixaban were identified with first exposures from Oct 19, 2010, i.e. the date dabigatran was approved in the U.S., through Oct 1, 2015 to provide at least 90 days of follow-up. The date of the first prescription filled of each medication was defined as the index date. Patients were required to have at least one inpatient or two outpatient diagnoses of NVAF within 60-days before the index date based on the International Classification of Diseases, Ninth Revision (ICD-9) code 427.31. Patients were included if they had at least 12-months of continuous enrollment before the index date (defined as the baseline period), and at least 3-months of continuous enrollment in the health plan after the index date to assess medication adherence. For each subsequent medication adherence interval (6-, 9-, and 12-months), patients were included only if they had continuous eligibility during the whole interval. Patients were excluded if they had prescription for any of the studied medications or warfarin during the baseline period to ensure an anticoagulant treatment-naïve cohort. We also excluded patients with more than one oral anticoagulant (OAC) on the index date, those with a diagnosis of mitral valve disease, heart valve repair or replacement, or joint replacement during the baseline period, consistent with exclusion criteria for pivotal clinical trials and indicated uses for DOACs.

### Medication use

Prescription fills of warfarin and DOACs (dabigatran, rivaroxaban, and apixaban) were identified by active pharmaceutical ingredient. Use of these medications each day during follow up was assessed using the refill date and days supplied variables on the claim. The first medication used after diagnosis was considered the index medication. When there was an overlap in dispensing for the same medication (patients refilled the medication before the original prescription period was completed), the new refill was assumed to begin the day after the end of the previous dispensing. If overlap occurred when patients switched to a new medication, the new medication was assumed to be initiated on that dispensing date.

### Adherence measure

The proportion of days covered (PDC) was used to measure adherence. PDC was calculated by dividing the total number of days that the patients had the medication by the follow up period. In our study, we evaluated adherence at 3, 6, 9 and 12- month intervals. We also calculated the PDC to any OAC in case patient switched to another medication to distinguish if patients experienced treatment interruptions due to the switch. For example, if a patient initiated apixaban for the first 30 days during 3-month follow-up, then switched to warfarin at day 31 and continued to fill a total of 60 days supplied of warfarin, the PDC for apixaban would be 0.33 (30 days/90 days) and the PDC for any OAC would be 1.0 (30 days of apixaban+ 60 days of warfarin/90 days). We also evaluated therapy gaps and switches during follow up. Gaps were identified as at least ≥3, ≥7, and ≥ 15 days. For switching, the type of the medication that the patients switched to was reported. Proportions of patients having these gaps or switches were reported in each interval.

### Study variables

Baseline characteristics assessed during the 1-year pre-index period included patient demographic (age, sex, geographic region), medical history (diabetes, hypertension, hyperlipidemia, heart failure, stroke, bleeding, renal disease, liver disease, dementia, vascular disease, chronic pulmonary disease, rheumatic disease, cancer, metastatic cancer, smoking, Charlson Comorbidity Index (CCI), CHA_2_DS_2_-VASc Score, HAS-BLED Score) and health care utilization (number of outpatient visits, number of medication used, insurance plan type) and medication use. Stroke risk score (CHA_2_DS_2_-VASc Score) was calculated based on specific comorbidities by adding 1 point each for congestive heart failure, hypertension, diabetes, vascular disease, age (65–74), female sex, and 2 points each for cerebrovascular disease or age ≥ 75 [18]. The maximum score for CHA_2_DS_2_-VASc Score is 9 points. Bleeding risk was also evaluated using HAS-BLED Score [19]. Each of these conditions including hypertension, abnormal renal function, stroke, bleeding history or predisposition, elderly (≥65), antiplatelet or non-steroidal anti-inflammatory drugs (NSAIDs) use and alcoholism was assigned 1 point. We did not include the “labile international normalized ratio” in the HAS-BLED calculation due to the cohort being treatment naïve; thus, the maximum score for our modified HAS-BLED score was 8 instead of 9. The number of days supplied on the first refill and the use of mail or retail pharmacies was also assessed.

### Statistical analysis

Baseline characteristics were reported as proportions for categorical variables and means with standard deviation for continuous variables. Analysis of variance and chi-square tests were used to compared PDC between treatment groups. Adherence was evaluated when stratified by CHA_2_DS_2_-VASc score, the days supplied of the first fill (30 days or ≥ 30 days), and use of retail or mail pharmacy.

A multivariable logistic regression model was used to compare the likelihood of high adherence (PDC ≥0.80) as a binary outcome among DOACs in each time interval and included all baseline characteristics. Adjusted odds ratios (OR) and 95% confidence intervals (95% CIs) were reported. All analyses were performed using SAS Version 9.4 (SAS Institute, Cary, NC).

## Results

### Summary treatment group characteristic

**Fig. 1:**
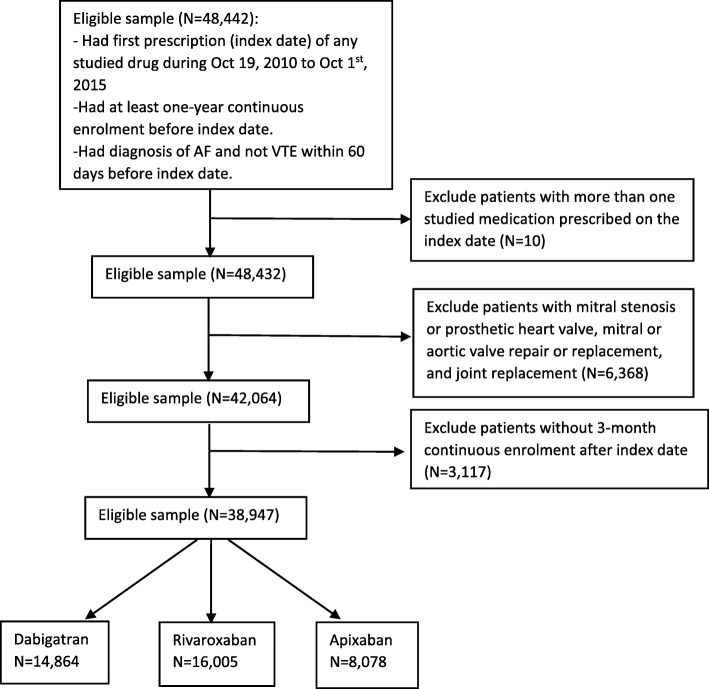
Cohort selection and attrition. Application of inclusion and exclusion criteria

There were 14,864 dabigatran users, 16,005 rivaroxaban users, and 8078 apixaban users identified during the study period (Fig. 1). The baseline characteristics of each treatment group are shown in Table 1. Apixaban users were older and had more comorbidities compared to rivaroxaban and dabigatran users, however, the mean CHA_2_DS_2_-VASc score and HAS-BLED score were similar across treatment groups.

**Table 1:** Baseline characteristics of dabigatran users, rivaroxaban users and apixaban users

Patient characteristics	Dabigatran *N* = 14,864	Rivaroxaban *N* = 16,005	Apixaban *N* = 8078
Demographic			
Age, N (%)			
< 65	7322 (49.3)	7563 (47.2)	3388 (41.9)
65- < 70	1613 (11.0)	1801 (11.2)	892 (11.0)
70- < 75	1640 (11.0)	1842 (11.5)	871 (10.8)
75- < 80	1659 (11.2)	1745 (10.9)	948 (11.7)
80- < 85	1480 (10.0)	1609 (10.0)	959 (11.9)
≥ 85	1131 (7.6)	1445 (9.0)	1020 (12.6)
Sex, N (%)			
Male	9126 (61.4)	9507 (59.4)	4579 (56.7)
Female	5738 (38.6)	6498 (40.6)	3499 (43.3)
Region			
Northeast	3291 (22.1)	3250 (20.3)	1554 (19.2)
North Central	3972 (26.7)	4622 (28.9)	2305 (28.5)
South	5259 (35.4)	5820 (36.4)	3301 (40.9)
West	2054 (13.8)	2096 (13.1)	838 (10.4)
Unknown	288 (1.9)	217 (1.4)	80 (1.0)
Medical history, N (%)			
Diabetes w/ complication	806 (5.4)	1051 (6.6)	628 (7.8)
Diabetes w/o complication	3688 (24.8)	4148 (25.9)	2134 (26.4)
Hypertension	10,517 (70.7)	11,933 (74.6)	6398 (79.2)
Hyperlipidemia	7798 (52.5)	9230 (57.7)	5037 (62.4)
Heart failure	2921 (19.6)	3312 (20.7)	1848 (22.9)
Stroke	657 (4.4)	794 (5.0)	506 (6.3)
Myocardial infarction	842 (5.7)	997 (6.2)	624 (7.7)
Bleeding	1209 (8.1)	1355 (8.5)	756 (9.4)
Renal disease	812 (5.5)	1075 (6.7)	819 (10.1)
Liver disease	473 (3.2)	559 (3.5)	308 (3.8)
Dementia	92 (0.6)	165 (1.0)	121 (1.5)
Vascular disease	5000 (33.6)	5476 (34.2)	3079 (38.1)
COPD	3120 (21.0)	3664 (22.9)	1936 (24.0)
Rheumatic disease	395 (2.7)	476 (3.0)	236 (2.9)
Peptic ulcer disease	80 (0.5)	79 (0.5)	47 (0.6)
Cancer	2109 (14.2)	2358 (14.7)	1328 (16.4)
Metastatic cancer	149 (1.0)	161 (1.0)	96 (1.2)
Smoking	1512 (10.2)	1862 (11.6)	1058 (13.1)
CCI, mean (SD)	1.6 (1.9)	1.7 (1.9)	1.9 (2.1)
CHA_2_DS_2_-VASc, mean (SD)	3.0 (1.9)	3.1 (2.0)	3.4 (2.0)
0–1	3654 (24.6)	3766 (23.5)	1495 (18.5)
2	2779 (18.7)	2977 (18.6)	1478 (18.3)
3	2585 (17.4)	2744 (17.1)	1386 (17.2)
≥ 4	5846 (39.3)	6518 (40.7)	3719 (46.0)
HAS-BLED, mean (SD)	1.8 (1.1)	1.9 (1.1)	2.0 (1.2)
0–1	6103 (41.1)	6159 (38.5)	2659 (32.9)
2	5084 (34.2)	5434 (33.9)	2828 (35.0)
3	2715 (18.3)	3201 (20.0)	1721 (21.3)
≥ 4	962 (6.5)	1211 (7.6)	870 (10.8)
Medication use, N (%)			
General			
Estrogen therapy	692 (4.7)	665 (4.1)	382 (4.7)
Histamine 2 antagonists	491 (3.3)	578 (3.6)	347 (4.3)
NSAID	3180 (21.4)	3676 (23.0)	1818 (22.5)
Proton pump inhibitors	3249 (21.9)	3803 (23.8)	2089 (25.9)
SSRI antidepressants	1638 (11.0)	1889 (11.8)	993 (12.3)
Cardiovascular			
ACE inhibitors	4932 (33.2)	5138 (32.1)	2741 (33.9)
ARBs	3126 (21.0)	3464 (21.6)	1836 (22.7)
Antiarrhymics	4334 (29.2)	4406 (27.5)	2276 (28.2)
Other anticoagulants	135 (0.9)	103 (0.6)	42 (0.5)
Antiplatelet	1735 (11.7)	1761 (11.0)	1060 (13.1)
Beta-blocker	5031 (33.8)	5268 (32.9)	2761 (34.2)
Calcium channel blocker	6244 (42.0)	6867 (42.9)	3567 (44.2)
Digoxin	2053 (13.8)	1832 (11.4)	823 (10.2)
Diuretics			
Loop	2765 (18.6)	2979 (18.6)	1697 (21.0)
Potassium sparing	1166 (7.8)	1198 (7.5)	632 (7.8)
Thiazide and other	4216 (28.4)	4489 (28.0)	2347 (29.0)
Nitrates	1044 (7.0)	1085 (6.8)	666 (8.2)
Statins	7129 (48.0)	7714 (48.2)	4111 (50.9)
Fibrates	649 (4.4)	678 (4.2)	315 (3.9)
Other antihyperlipidemic	1313 (8.8)	1135 (7.1)	554 (6.9)
Diabetes related			
Insulin	780 (5.2)	844 (5.3)	439 (5.4)
Metformin	2011 (13.5)	2275 (14.2)	1125 (13.9)
Sulfonylurea	1044 (7.0)	1071 (6.7)	560 (6.9)
Other	1271 (8.5)	1278 (8.0)	704 (8.7)
Metabolic inhibitors			
Amiodarone	1570 (10.6)	1780 (11.1)	969 (12.0)
Dronedarone	1345 (9.0)	900 (5.6)	496 (6.1)
Azole antifungals	2313 (15.6)	2569 (16.0)	1361 (16.8)
Verapamil	386 (2.6)	365 (2.3)	180 (2.2)
Diltiazem	3372 (22.7)	3786 (23.7)	1922 (23.8)
Metabolic inducers			
Carbamazepine	31 (0.2)	26 (0.2)	2 (0.3)
Phenytoin	37 (0.3)	29 (0.2)	14 (0.2)
Phenobarbital	13 (0.1)	12 (0.1)	4 (0.1)
Prescriber specialty			
Internal medicine	2273 (15.3)	2554 (16.0)	1107 (13.7)
Family practice	1526 (10.3)	1795 (11.2)	1006 (12.4)
Geriatric medicine	10 (0.1)	16 (0.1)	6 (0.1)
Cardiology	5666 (38.1)	5912 (36.9)	2965 (36.7)
Critical care medicine	10 (0.1)	12 (0.1)	6 (0.1)
Hematology	10 (0.1)	11 (0.1)	8 (0.1)
Oncology	7 (0.1)	13 (0.1)	7 (0.1)
Healthcare utilization			
Outpatient visits, N (%)			
≤ 9	4489 (30.2)	4794 (29.9)	2075 (25.7)
> 9–16	4009 (27.0)	4149 (25.9)	2191 (27.1)
> 16–29	3826 (25.7)	4214 (26.3)	2222 (27.5)
> 29	2540 (17.1)	2848 (17.8)	1590 (19.7)
Inpatient visits, mean (SD)	0.67 (1.71)	0.64 (0.69)	0.61 (0.71)
Emergency visits, mean (SD)	0.51 (1.03)	0.60 (1.21)	0.65 (1.18)
Number of Rx, N (%)			
≤ 12	3373 (22.7)	3637 (22.7)	1774 (22.0)
> 12–18	3943 (26.5)	4113 (25.7)	2115 (26.2)
> 18–25	3897 (26.2)	4221 (26.4)	2189 (27.1)
> 25	3651 (24.6)	4034 (25.2)	2000 (24.8)
Plan type			
HMO	1597 (11.3)	1803 (11.5)	845 (10.7)
Non-HMO	13,267 (88.7)	14,202 (88.5)	7233 (89.3)
Number of first day supply			
≤ 30 days	12,967 (87.2)	14,046 (87.8)	6776 (83.9)
> 30 days	1897 (12.8)	1959 (12.2)	1302 (16.1)
First fill method			
Via retail pharmacy	12,844 (89.2)	14,633 (92.8)	7127 (89.5)
Via mail	1555 (10.8)	1135 (7.2)	838 (10.5)
Enrollment period			
6 months continuous	13,877 (93.4)	14,090 (88.0)	6438 (79.7)
9 months continuous	12,866 (86.6)	12,112 (75.7)	4943 (61.2)
12 months continuous	11,689 (78.6)	10,195 (63.7)	3500 (43.3)

*COPD* chronic obstructive pulmonary disease, *CCI* Charlson Comorbidities Index, *NSAID* nonsteroidal anti-inflammatory drug, *SSRI* selective serotonin reuptake inhibitor, *ACE inhibitor* Angiotensin-converting enzyme inhibitor, *ARBs* Angiotensin II receptor blockers, *HMO* Health Maintenance Organization

### Adherence to treatment

At 3-month follow-up, mean PDC for apixaban users was 0.83, following by rivaroxaban (0.81) and dabigatran (0.78) (*p* < 0.001; Table 2). Apixaban also had the highest proportion of patients with PDC ≥ 80 (70.25%). Similar rankings between DOACs were observed at 6, 9 and 12-month follow up. At 12-months, mean PDC for apixaban, rivaroxaban and dabigatran went down to 0.70, 0.64 and 0.57, respectively.

**Table 2:** Proportion of Days Covered (PDC) by index medication, switching pattern and medication gap among DOAC users at 3, 6, 9 and 12 months of follow-up

	Dabigatran	Rivaroxaban	Apixaban	*P*-value
3 months				
PDC, mean (SD)	0.78 (0.27)	0.81 (0.27)	0.83 (0.25)	<.0001
≥ 0.8, n (%)	9196 (61.87)	10,865 (67.89)	5675 (70.25)	
0.5–.079, n (%)	2495 (16.79)	2188 (13.67)	1199 (14.84)	
< 0.5, n (%)	3173 (21.35)	2952 (18.44)	1204 (14.90)	
Switching, n (%)	1016 (6.84)	729 (4.55)	255 (3.16)	<.0001
*Warfarin*	611 (4.11)	414 (2.59)	118 (1.46)	
*Dabigatran*	–	101 (0.63)	23 (0.28)	
*Rivaroxaban*	341 (2.29)	–	114 (1.41)	
*Apixaban*	64 (0.43)	214 (1.34)	–	
Gap, n (%)				
*≥ 3 days*	7276 (48.95)	7055 (44.08)	3662 (45.33)	<.0001
*≥ 7 days*	6026 (40.54)	5770 (36.05)	2892 (35.80)	<.0001
*≥ 15 days*	4953 (33.32)	4689 (29.30)	2262 (28.00)	<.0001
6 months				
PDC, mean (SD)	0.67 (0.33)	0.72 (0.32)	0.76 (0.29)	
≥ 0.8, n (%)	7156 (52.57)	8241 (58.49)	4054 (62.97)	
0.5–.079, n (%)	2698 (19.44)	2491 (17.68)	1189 (18.47)	
< 0.5, n (%)	4023 (28.99)	3358 (23.83)	1195 (18.56)	
Switching, n (%)	1369 (9.87)	936 (6.64)	321 (4.99)	<.0001
*Warfarin*	778 (5.61)	523 (3.71)	155 (2.41)	
*Dabigatran*	–	124 (0.88)	24 (0.37)	
*Rivaroxaban*	501 (3.61)	–	142 (2.21)	
*Apixaban*	90 (0.65)	289 (2.05)	–	
Gap, n (%)				
*≥ 3 days*	9511 (68.54)	8924 (63.34)	4230 (65.70)	<.0001
*≥ 7 days*	8334 (60.06)	7645 (54.26)	3520 (54.68)	<.0001
*≥ 15 days*	7029 (50.65)	6402 (45.44)	2829 (43.94)	<.0001
9 months				
PDC, mean (SD)	0.61 (0.35)	0.67 (0.34)	0.72 (0.31)	<.0001
≥ 0.8, n (%)	5579 (43.36)	6260 (51.68)	2852 (57.70)	
0.5–.079, n (%)	2193 (17.04)	1818 (15.01)	839 (16.97)	
< 0.5, n (%)	5094 (39.59)	4034 (33.31)	1252 (25.33)	
Switching, n (%)	1590 (12.36)	990 (8.17)	299 (6.05)	<.0001
*Warfarin*	884 (6.87)	559 (4.62)	137 (2.77)	
*Dabigatran*	–	121 (1.00)	23 (0.47)	
*Rivaroxaban*	600 (4.66)	–	139 (2.81)	
*Apixaban*	106 (0.82)	310 (2.56)	–	
Gap, n (%)				
*≥ 3 days*	9723 (75.57)	8593 (70.95)	3607 (72.97)	<.0001
*≥ 7 days*	8709 (67.69)	7553 (62.36)	3090 (62.51)	<.0001
*≥ 15 days*	7528 (58.51)	6445 (53.21)	2545 (51.49)	<.0001
12 months				
PDC, mean (SD)	0.57 (0.36)	0.64 (0.36)	0.70 (0.33)	<.0001
≥ 0.8, n (%)	4761 (40.73)	5080 (49.83)	1969 (56.26)	
0.5–.079, n (%)	1587 (13.58)	1284 (12.59)	498 (14.23)	
< 0.5, n (%)	5314 (45.69)	3831 (37.58)	1033 (29.51)	
Switching, n (%)	1717 (14.69)	559 (9.41)	263 (7.51)	<.0001
*Warfarin*	930 (7.96)	545 (5.35)	121 (3.46)	
*Dabigatran*	–	109 (1.07)	19 (0.54)	
*Rivaroxaban*	663 (5.67)	–	123 (3.51)	
*Apixaban*	124 (1.06)	305 (2.99)	–	
Gap, n (%)				
*≥ 3 days*	9350 (79.99)	7771 (76.22)	2741 (78.31)	<.0001
*≥ 7 days*	8475 (72.50)	6850 (67.19)	2331 (66.60)	<.0001
*≥ 15 days*	7428 (63.55)	5936 (58.22)	1980 (56.57)	<.0001

*PDC* proportion of days covered, *SD* standard deviation

Roughly 5–7% of patients switched to another OAC after 3-month and increased up to about 8–15% after 12-month. Dabigatran had higher proportions of users switching to other treatments compared to rivaroxaban and apixaban. Switching options also differed between DOACs. Warfarin was the preferred choice for those switching from dabigatran and rivaroxaban while apixaban users tended to switch to another DOAC. Dabigatran users also had a higher rate of treatment gaps compared to both rivaroxaban and apixaban (Table 2).

Adherence differed when stratified by stroke risk scores. Mean PDC among patients with CHA_2_DS_2_-VASc score ≥ 4 was above 0.70 while it was less than 0.55 among those with CHA_2_DS_2_-VASc score ≤ 1 (Table 3). PDC was much higher with 90-day supply (dabigatran: 0.72, rivaroxaban: 0.79, apixaban: 0.84) versus 30-day supply (dabigatran 0.55, rivaroxaban: 0.63, apixaban: 0.66), and higher when filled via mail pharmacy (dabigatran: 0.71, rivaroxaban: 0.79, apixaban: 0.84) compared to retail pharmacy (dabigatran: 0.55, rivaroxaban: 0.63, apixaban: 0.67) (Table 4).

**Table 3:** Proportion of Days Covered (PDC) among DOAC users by index medication and by any OAC stratified by stroke risk score (CHA_2_DS_2_-VASc) at 12 months of follow-up

PDC, mean (SD)	Dabigatran (*n* = 11,650)	Rivaroxaban (*n* = 10,156)	Apixaban (*n* = 3486)	*P*-value
Adherence to index DOAC				
Overall	0.57 (0.36)	0.64 (0.36)	0.70 (0.33)	
CHA_2_DS_2_-VASc ≤1	0.44 (0.34)	0.49 (0.35)	0.52 (0.35)	<.0001
CHA_2_DS_2_-VASc = 2	0.56 (0.35)	0.64 (0.35)	0.68 (0.33)	<.0001
CHA_2_DS_2_-VASc = 3	0.62 (0.35)	0.69 (0.34)	0.73 (0.32)	<.0001
CHA_2_DS_2_-VASc ≥4	0.64 (0.35)	0.70 (0.34)	0.76 (0.30)	<.0001
Adherence to any OAC				
Overall	0.64 (0.34)	0.68 (0.34)	0.73 (0.31)	
CHA_2_DS_2_-VASc ≤1	0.47 (0.34)	0.52 (0.35)	0.54 (0.36)	<.0001
CHA_2_DS_2_-VASc = 2	0.62 (0.34)	0.67 (0.34)	0.71 (0.32)	<.0001
CHA_2_DS_2_-VASc = 3	0.68 (0.33)	0.73 (0.32)	0.77 (0.28)	<.0001
CHA_2_DS_2_-VASc ≥4	0.72 (0.31)	0.75 (0.31)	0.80 (0.27)	<.0001

*DOAC* direct-acting oral anticoagulant, *OAC* oral anticoagulant

**Table 4:** Adherence comparison to index medication among DOAC users stratified by first day supply and fill method at 12 months of follow-up

	Dabigatran	Rivaroxaban	Apixaban	*P*-value
First day supply = 30 days				
PDC, mean (SD)	0.55 (0.36)	0.63 (0.36)	0.66 (0.34)	<.0001
≥0.8, n (%)	3852 (38.56)	4036 (48.29)	1424 (52.60)	
0.5–.079, n (%)	1349 (13.60)	1036 (12.40)	396 (14.63)	
< 0.5, n (%)	4746 (47.84)	3285 (39.31)	887 (32.77)	
First day supply = 90 days				
PDC, mean (SD)	0.72 (0.30)	0.79 (0.28)	0.84 (0.24)	<.0001
≥0.8, n (%)	845 (56.48)	824 (66.83)	458 (73.87)	
0.5–.079, n (%)	198 (13.24)	147 (11.92)	73 (11.77)	
< 0.5, n (%)	453 (30.28)	262 (21.25)	89 (14.35)	
Filled via mail pharmacy (1st fill)				
PDC, mean (SD)	0.71 (0.31)	0.79 (0.28)	0.84 (0.24)	<.0001
≥0.8, n (%)	755 (56.72)	509 (66.10)	328 (73.71)	
0.5–.079, n (%)	169 (12.70)	100 (12.99)	58 (13.03)	
< 0.5, n (%)	407 (30.58)	161 (20.91)	59 (13.26)	
Filled via retail pharmacy (1st fill)				
PDC, mean (SD)	0.55 (0.36)	0.63 (0.36)	0.67 (0.34)	<.0001
≥0.8, n (%)	3907 (38.70)	4522 (48.59)	1616 (53.62)	
0.5–.079, n (%)	1377 (13.64)	1164 (12.51)	439 (14.57)	
< 0.5, n (%)	4811 (47.66)	3620 (38.90)	959 (31.82)	

*PDC* proportion of days covered, *SD* standard deviation

### Regression results

Multivariable logistic models were used to compare the likelihood of having high adherence (PDC ≥0.8) among DOAC users after controlling for other patient baseline factors. Dabigatran users had roughly 30% lower odds of being highly adherent to therapy compared to other DOACs during each time interval (Table 5). High adherence for rivaroxaban versus dabigatran did not change much over time (OR = 1.34 at 3-months and OR = 1.40 at 12-months), it increased significantly for apixaban versus dabigatran (OR = 1.41 at 3-month to OR = 1.73 at 12-month). No difference was found between adherence to apixaban and rivaroxaban at 3-month but, beginning at the 6-month interval, apixaban users were more likely to have high adherence compared to rivaroxaban. Similar patterns was observed for adherence to any OACs as apixaban users had the highest odds of high adherence while dabigatran users had the lowest. Difference between the drugs was less pronounced for overall OAC adherence than adherence to the index DOAC.

**Table 5:** Adjusted Odds Ratio for high adherence to index OAC and to any OAC during 3, 6, 9 and 12 months of follow up

	3 months		6 months		9 months		12 months	
	Adj OR	95% CI	Adj OR	95% CI	Adj OR	95% CI	Adj OR	95% CI
PDC ≥ 0.80 to index OAC								
R vs. D	1.34	1.27–1.40*	1.32	1.25–1.39*	1.37	1.30–1.44*	1.40	1.32–1.48*
A vs. D	1.41	1.33–1.50*	1.52	1.42–1.62*	1.67	1.56–1.79*	1.73	1.60–1.88*
A vs. R	1.06	0.99–1.12	1.15	1.08–1.23*	1.22	1.14–1.31*	1.24	1.14–1.34*
PDC ≥ 0.80 to any OAC								
R vs. D	1.26	1.20–1.33*	1.23	1.17–1.29*	1.29	1.23–1.36*	1.32	1.25–1.40*
A vs. D	1.28	1.20–1.36*	1.40	1.31–1.49*	1.55	1.44–1.66*	1.62	1.50–1.76*
A vs. R	1.01	0.95–1.08	1.13	1.06–1.21*	1.20	1.12–1.29*	1.23	1.13–1.34*

*DOAC* direct-acting oral anticoagulant, *OAC* oral anticoagulant, *R* rivaroxaban, *D* dabigatran, *A* apixaban, *OR* odds ratio, *CI* confidence interval, *PDC* proportion of days covered

*denotes statistical significance at *P* <0.05

## Discussion

Among the commercially insured NVAF patients in the U.S., this study found consistently poorer adherence for dabigatran compared to rivaroxaban and apixaban, which has not changed compared to prior studies. However, there was a signficant alteration in the adherence results observed for rivaroxaban when compared to apixaban. Results suggest that rivaroxaban, which has had shown a slight advantage in adherence in past studies [16, 17], did not impart higher adherence despite its once daily dosing schedule compared to the twice-daily regimen of apixaban. These findings persisted for the overall cohort as well as subgroups of patients at high and very high risk of stroke according to baseline CHA_2_DS_2_-VASc scores. In fact, adherence to any OAC treatment was consistently higher for apixaban users versus other treatments, which is especially important among those with CHA_2_DS_2_-VASc ≥2 as guidelines routinely recommend long-term OAC for stroke prevention [20, 21]. Measuring PDC to any OAC takes into account switching between DOACs or to warfarin and serves as a proxy measure of overall treatment adherence and concordance with treatment guidelines [20].

Our findings are generally consistent with prior studies, but these studies may differ in how inclusion and exclusion criteria are defined and how adherence is measured. A recent study by Crivera et al. assessed PDC to DOACs in a large managed care database [13]. They found that around 75% of rivaroxaban, 70% of apixaban, and 67% of dabigatran users were highly adherent to therapy over a 1-year period. These values are higher than the proportions found in this study and also showed a larger difference between rivaroxaban and apixaban. Their methodology used a distinct algorithm for identifying users according to standardized methods and was also not specific to NVAF as they considered DOAC adherence across all indications. Their findings are consistent with the current study, however, showing dabigatran with lower overall adherence compared to both comparators [13]. The current study updated prior studies by our group using identical approaches; thus serves as a consistent indicator of changing patterns in adherence for this patient population [16, 17]. Notable changes in the current study suggest changing trends in adherence as apixaban users had higher adherence to rivaroxaban in updated results but were lower or identical in adherence in prior studies. The implications and drivers of this phenomenon are unknown and deserve further study but may be related to the higher minor bleed rates associated with rivaroxaban which may lead to more non-adherence and switching compared to apixaban [6–8].

Adherence to anticoagulation is pivotal to prevent stroke associated with NVAF [22–25]. Although overall adherence has not been definitively shown to be associated with stroke risk, gaps in therapy during treatment with warfarin has been shown to impart increased risk of stroke [25]. Gaps in therapy can include missed doses as well as delays in filling subsequent prescriptions. While individual missed doses could not be assessed in these data, we did observe the number of gaps for each treatment group and showed dabigatran had more gaps than the other therapies. While we assessed longer gaps of 3, 7, and 15 days, even small gaps due to single missed doses while on DOACs can be high risk considering the short half-lives of these medications [9]. Thus, while the choice of initial DOAC did not dramatically influence overall adherence to OAC, small interruptions in therapy could still be detrimental for clinical outcomes.

Applying these findings in a clinical framework, identifying the most efficacious medication is moot if patients discontinue therapy in a matter of months or have multiple treatment gaps thereby leading to the drug not being effective in real-world settings. As mentioned, comparative effectiveness studies have generally concluded a net clinical benefit ranking of apixaban, dabigatran, and then rivaroxaban. However, high utilization of rivaroxaban use has persisted, likely due to the assumption of better adherence with once-daily treatment options, which is not supported by the results of our study. While the trade-off between dosing schedule and effectiveness may be important considerations for prescribers, for patients a recent conjoint analysis showed that effectiveness and safety considerations are an order of magnitude more important to patients than dosing schedules [26]. Incorporating real-world patient behaviors including adherence, persistence, compliance, and switching is important to inform clinicians and decision makers of the real-world effectiveness of these therapies. Future comparative studies should incorporate time-varying confounding through more advanced modeling to account for the influence censoring, and other study design aspects, may have had on that study [27].

Adherence to DOACs may further become an important policy issue for managed care companies in the United States as the PQA has developed a quality metric for this measure. PQA adherence measures exist for other medication classes for diabetes and hypercholesterolemia and have been incorporated as standardized metrics to compare health plan and physician quality. For Medicare Part D and Medicare Advantage plans, these measure are given a significant weight in the Star Ratings calculation [28]. Given enrollee selection of plans and reimbursement are tied to these Star Ratings, health plans are strongly incentivized to develop interventions to increase their ratings, including enrollee-directed interventions or formulary management decisions [29, 30]. Further, in practices where payment is tied to provider performance, these measures may also impact prescribers and make our study more important to help guide prescribing choices. It is encouraging that treatment rates among NVAF patients have increased since the introduction of DOACs on the market [31]. New efforts to focus on and improve treatment adherence with continued increasing treatment rates concordant with guideline recommendations are needed [32].

### Limitations

This study is subject to the limitations of all claims-based studies [33, 34]. Notably for this adherence study, the data captured prescription utilization and assumed that a patient consumed the medication and was compliant with the dosing regimen, although this cannot be confirmed. We did not incorporate patients who initiated warfarin as it is well understood that warfarin therapy is wrought with high discontinuation rates and poor adherence and utilization may also be missing in claims-based studies [35, 36]. Further, there are more differences between warfarin and DOAC users in the post-DOAC era, which could have potentially biased these results [31]. Measures related to 90-day days supplied and mail order pharmacies may have influenced the results and were included as important factors to consider when measuring adherence in claims data. Longer days supplied will increase adherence when calculated via PDC but may miss non-adherence when patients discontinue in the midst of this supply period. Likewise, mail order services are often “auto-refill” services which will fill prescriptions without patient involvement; thus ignoring patient behaviors. For the purposes of this analysis, we sought to control for these factors and expect they would have non-differential influences on our results and the comparisons made. PDC is also an imperfect measure of adherence as it cannot capture patient adherence within a days supplied period and does not capture reasons for non-adherence. However, on a macro-level, PDC has been associated with quality of care and shown to generally correspond with other direct measures of patient adherence to therapy for NVAF, although direct and indirect adherence measures may capture different domains of patient behavior [37].

Our patient selection involved four time periods of continuous eligibility to be included in the analysis. This was done to have standardized follow-up times between patients and reduce the impact of time-varying confounding on the adherence measures [38]. This will mostly remove those who die during follow-up as well as those who disenroll from their health plan. Thus, the measure of adherence will not account for these groups of patients [39]. However, previous studies have shown that those who die during initial treatment of NVAF have similar adherence to those that survive [40]. Finally, the study findings are applicable for the commercially insured or those with Medicare supplemental retiree coverage and may not generalizable to the uninsured or patients with public or government insurance, such as those with Medicare fee-for-service.

## Conclusions

In this study of newly diagnosed, treatment-naïve NVAF patients, those initiating anticoagulation with dabigatran had lower adherence and more switching during 3, 6, 9 and 12 months of follow-up compared to rivaroxaban and apixaban users. More importantly, apixaban, a twice-daily medication, had better overall adherence than rivaroxaban, which is a once-daily medication. As adherence to DOACs is important for treatment success and is a potential future quality of care indicator, managed care plans and prescribers should be aware of the differences in adherence between treatment options in addition to the evidence for safety and efficacy.

## Abbreviations

CI: Confidence interval
DOAC: Direct oral anticoagulant
NVAF: Non-valvular atrial fibrillation
OAC: Oral anticoagulant
PDC: Proportion of days covered
PQA: Pharmacy Quality Alliance CMS Centers for Medicare and Medicaid Services
